# EUS-Guided Pancreatic Cyst Ablation: a Clinical and Technical Review

**DOI:** 10.1007/s11894-019-0686-5

**Published:** 2019-04-23

**Authors:** Matthew T. Moyer, Jennifer L. Maranki, John M. DeWitt

**Affiliations:** 1 Division of Gastroenterology and Hepatology, Penn State Cancer Institute, Penn State Hershey Medical Center, Hershey, PA, USA; 2 Division of Gastroenterology and Hepatology, Indiana University School of Medicine, Indianapolis, IN, USA

**Keywords:** Pancreatic cyst treatment, Pancreatic cyst ablation, Pancreatic cancer prevention

## Abstract

**Purpose of Review:**

Pancreatic cystic lesions represent a growing public health dilemma, particularly as our population ages and cross-sectional imaging becomes more sensitive. Mucinous cysts carry a clinically significant risk of developing pancreatic cancer, which carries an extremely poor prognosis. Determining which cysts will develop cancer may be challenging, and surgical resection of the pancreas carries significant morbidity. The goal of this paper is to review the rationale for cyst ablation and discuss prior and current research on cyst ablation techniques and efficacy. Indications, contraindications, and factors related to optimal patient selection are outlined.

**Recent Findings:**

Endoscopic ultrasound-guided chemoablation of pancreatic cysts has been performed in neoplastic cysts, with varying levels of efficacy. Safety concerns arose due to the risk of pancreatitis in alcohol-based treatments; however, the most recent data using a non-alcohol chemoablation cocktail suggests that ablation is effective without the need for alcohol, resulting in a significantly more favorable adverse event profile.

**Summary:**

Endoscopic ultrasound-guided chemoablation of neoplastic pancreatic cysts is a promising, minimally invasive approach for treatment of cysts, with recent significant advances in safety and efficacy, suggesting that it should play a role in the treatment algorithm.

## Background

Over 55,000 people will develop pancreatic cancer this year in the United States, with 95% of cases being fatal, making it the fourth leading cause of cancer-associated mortality [1–3]. As a lethal malignancy with a dismal 5-year survival rate of 8%, it has the lowest survival rate of any major cancer and is projected to surpass colorectal cancer as the second leading cause of cancer-related death by 2030 without breakthroughs in prevention and therapy as we have seen in colon cancer [1–3]. While the majority of pancreatic cancers are ductal adenocarcinomas, approximately 20% of pancreatic cancer develops from mucinous-type pancreatic cysts, and this percentage may be underreported [2, 4]. Pancreatic cystic lesions are typically discovered incidentally on cross-sectional imaging, occurring in approximately 2% of American adults, with a 37% prevalence in individuals older than 80 years old [5]. The prevalence of pancreatic cysts in the United States has grown dramatically over the last two decades due to an aging population and advances in imaging techniques, leading to a public health dilemma. Although certain types of pancreatic cysts carry little to no malignant potential, the majority are neoplastic and includes mucinous cystic neoplasm (MCN) and intraductal papillary mucinous neoplasm (IPMN), which carry a clinically significant potential for malignant transformation. The natural history of a mucinous pancreatic cyst is variable, with the overall risk of progression to pancreatic cancer progressing to invasive cancer generally linked to its number of high-risk features [6, 7•]. The overall risk of progression to malignancy for a mucinous cyst without high-risk features is reported to range from 1 to 25%, and it is difficult with current imaging techniques and cyst fluid analysis to discern which cyst will undergo malignant transformation [2, 8–10]. Molecular testing of cyst fluid may improve this risk stratification but is very expensive and requires further validation [7•, 11, 12]. Identification of a mucinous pancreatic cyst requires the clinician and patient to choose between indefinite radiographic surveillance (MRI or CT) or surgical resection, both of which have considerable limitations. Surveillance for malignancy carries significant economic and psychological burdens, and CT imaging includes some degree of radiation exposure and possible nephrotoxicity. On the other hand, surgical resection possesses a substantial risk for serious adverse events (20–40%) and mortality (1–5%) and still requires post-operative surveillance [13–15]. This clinical dilemma highlights an pressing need to develop effective, less expensive, and more minimally invasive approaches for this patient population.

In this respect, endoscopic ultrasound (EUS)-guided pancreatic cyst ablation has emerged as an innovative, promising minimally invasive approach for the treatment of neoplastic pancreatic cysts [16•, 17]. EUS-guided cyst ablation was first demonstrated by Gan et al. where 80% alcohol was infused into all types of pancreatic cysts (after cyst fluid aspiration) and lavaged for 3–5 min. Patients were then followed for 6 months. Overall, a complete response rate of 35% rate was found with a 0% risk of adverse events [18]. This study was followed by the prospective randomized EPIC trial, which demonstrated a 33% rate of complete cyst ablation after ethanol lavage with a serious adverse event rate of 4–5% (pancreatitis) [19]. To date, ten published studies have investigated the safety and efficacy of EUS-guided pancreatic cyst ablation using ethanol, only two of which used a randomized design. These studies are summarized in Table 1. Although ethanol ablation is feasible, the use of alcohol alone for pancreatic cyst ablation has consistently resulted in disappointing efficacy. These poor results were illustrated most recently in a prospective trial by Gomez et al. which found a dismal 9% rate of complete ablation and a 4% risk of pancreatitis [22]. Overall, the clinical value of pancreatic cyst ablation with ethanol alone is unfavorable due to the suboptimal response and significant adverse event rates and its use as a single agent should be abandoned.

Oh et al. added the innovative step of infusing and leaving paclitaxel within the pancreatic cyst after ethanol lavage [23]. The addition of paclitaxel (a chemotherapeutic agent that arrests cellular microtubule assembly and mitosis) has been shown in separate trials to raise complete ablation rates to 50–79% (Table 1). The significant increase in efficacy of ethanol lavage plus paclitaxel infusion compared to ethanol alone has resulted in this combination therapy becoming the preferred approach for pancreatic cyst ablation today and offering complete ablation rates similar to that seen in other ablative strategies in gastroenterology.

Despite this increase of efficacy, a significant limitation of alcohol ablation with or without paclitaxel for pancreatic cyst ablation has been the associated serious adverse events of pancreatitis, peritonitis, and venous thrombosis in 0–10% of patients [18–26, 27••]. Importantly, the mechanism for these reported complications is believed to be secondary to the potent inflammatory and toxic effects of alcohol on the surrounding pancreatic parenchyma and/or neighboring vessels. The recently published prospective, randomized, double-blind ChARM trial evaluated a completely alcohol-free chemoablation approach [27••]. In this study, 39 patients were randomized to saline cyst lavage or conventional alcohol lavage with both arms then treated with a chemotherapeutic cocktail tailored specifically for pancreatic neoplasia (38 mg gemcitabine + 6 mg/mL paclitaxel). At one year post-treatment, 61% of patients in the alcohol arm achieved complete ablation compared with 67% in the alcohol-free arm. These findings suggest that alcohol is not required for effective pancreatic cyst ablation when a chemotherapy cocktail specifically designed for pancreatic neoplasia is used. More importantly, the rate of adverse events was significantly lower in the alcohol-free arm (*p* = .01) as all minor and serious adverse events occurred in the alcohol arm. Minor adverse events were comprised entirely of abdominal pain, occurring in four patients in the alcohol arm (22%) and in zero patients in the alcohol-free arm. Collectively, these findings demonstrate that removal of alcohol from pancreatic cyst ablation reduces adverse events to that comparable to EUS-FNA. This is an important development, since significantly improving the risk profile of the procedure, while preserving its efficacy, increases the attractiveness of this therapeutic option for patients with pancreatic cysts. This trial has led to a larger prospective, randomized, NIH-funded, multicenter trial using a larger sample size and several technical improvements to the procedure with the results expected in four years [28].

An important metric of the ablative approach is the treatment durability over time. Two trials to date have addressed this, with DeWitt et al. demonstrating the long-term durability of pancreatic cyst ablation at two years, showing that the majority of patients who have an effective ablation will also have elimination of detectable KRAS mutations [26]. In a recent large, prospective, follow-up study of 164 patients undergoing EUS-guided ablation with alcohol followed by paclitaxel, Choi et al. demonstrated that when patients achieved complete EUS-guided pancreatic cyst ablation, 98.3% remained in remission at six-year follow-up, demonstrating an excellent durable response following ablative therapy [29].

Multiple areas of uncertainty remain with this approach, including demonstration of clinical reduction in incidence rates of pancreatic adenocarcinoma in appropriately matched groups, a better understanding of which patients are best treated with this approach, and an evidence-based demonstration of the financial profile of this approach compared with a surveillance and/or surgical strategy. An international white paper addressing some of these issues is expected to be published in the coming year.

## Patient Selection

If EUS-guided cyst ablation is to add significant clinical value; it should not be performed on certain high-risk lesions which are unlikely to fully respond to treatment. It should also be avoided in small, low-risk pancreatic cysts with little chance of malignant progression as they are more appropriate for routine surveillance. Ablative strategies should be focused on cystic tumors which are technically amendable to cyst ablation and do not show overt signs of malignancy.

Only cysts with a likelihood of progression to malignancy are currently candidates for ablation. For that reason, the first step is to use clinical, radiographic, cytologic, and chemical analysis by EUS-FNA to diagnose and risk stratify a mucinous cyst [30]. There remains a level of uncertainty in the accurate diagnosis of pancreatic cyst type in 2019, and further investigation is required to improve this accuracy. Although 2% of the US population will have a pancreatic cyst, the estimated prevalence of cysts > 2 cm is estimated at only 0.8% [31]. In a recent large study evaluating the long-term risk of pancreatic malignancy in patients with mucinous pancreatic cysts, 577 patients with presumed branch duct IPMN under surveillance at Massachusetts General Hospital were evaluated for a primary outcome of risk for malignancy over five years, compared to the US population. The overall rate of development of malignancy was 5.5%; however, of patients with cysts 1.5 cm or smaller, the overall risk of malignant transformation was 0.9%, demonstrating a very low risk for progression to malignancy for mucinous cysts < 1.5cm without other high-risk features [32]. Additionally, it is technically challenging to perform EUS-guided pancreatic cyst ablation on cysts < 1.5 cm, as there is 0.8cc of volume within the FNA needle itself, which prohibits meaningful exchange of chemotherapeutic injectate. Consequently, cyst size ranging from 1.5 to 5 cm is the typical range of pancreatic cysts treated in most trials to date [16•, 17]. Cyst morphology is an important consideration. Multiseptated cysts present a unique challenge to ablation since treatment must ensure that each loculation of the cyst is injected with the ablation agent. Although cysts can appear multiseptated on MRI-MRCP, EUS-FNA may collapse the entire cyst, indicating communication between these individual compartments which may be not appreciated on cross-sectional imaging. Most trials to date have favored unilocular to oligolocular cyst morphology and have avoided cyst ablation in lesions with more than 4–5 discrete cell chambers [16•].

When reviewing previous trials and the experience of our centers, the recommended ideal indications and contraindications for pancreatic cyst ablation can be used to guide therapy and are summarized as the following:

## Indications

Patients with previously identified pancreatic cysts from 1.5 to 5 cm in diameter which are consistent with mucinous-type cysts as per guidelines [33] including indeterminate-type cysts.

1. Contraindications:
  - Pregnancy
  - Similar to colonoscopy/polypectomy, the patient should have a reasonable 3–5-year life expectancy in order to benefit from treating a precancerous lesion
  - Inability to safely undergo a 30–60 min procedure with monitored anesthesia
  - Signs of malignancy or cytology suspicious for malignancy
  - Lesions which are consistent with a benign cyst by clinical, radiographic, cytologic, and chemical analysis (pseudocyst, serous cystadenoma, lymphangioma, duplication cyst) [30].
2. Relative contraindications:
  Cysts with the following high-risk features:
  - Main pancreatic duct dilation > 5 mm
  - Epithelial-type mural nodules
  - Pathologically thick-walls or septations
  - Cytology showing high-grade dysplasia.
  - Signs of common bile duct or pancreatic duct obstruction, solid mass component within or associated with a cyst, pathologic lymphadenopathy associated with the cyst, pancreatic duct stricture associated with tail atrophy.
  - Septated cysts with > 4–5 discrete individual compartments
  - Irreversible coagulopathy, neutropenia, or thrombocytopenia with platelets < 30,000
  - Of note, the following high-risk features are not considered to be a contraindication to ablation: growth in size, atypical cells on cytology, or symptoms referable to the pancreas.

We recommend that patients considered for EUS-guided pancreatic cyst ablation should undergo a full evaluation in the outpatient clinic setting, where their clinical, radiographic, and endoscopic characteristics can be reviewed and explained to the patient. Important factors to discuss include the natural history of these tumors, areas of uncertainty, and conventional options of surveillance and/or surgical resection as appropriate. A detailed informed consent is mandatory. It is the recommendation of a soon-to-be-published international white paper that these procedures be performed by interventional endoscopists with formal training and credentialing in EUS and familiar with interventional EUS procedures as previously described [34]. Additionally, these procedures should be performed in a high-volume referral center in a multidisciplinary setting.

### Cyst Ablation Technique

#### Technical Aspects of the Procedure

**Fig. 1:**
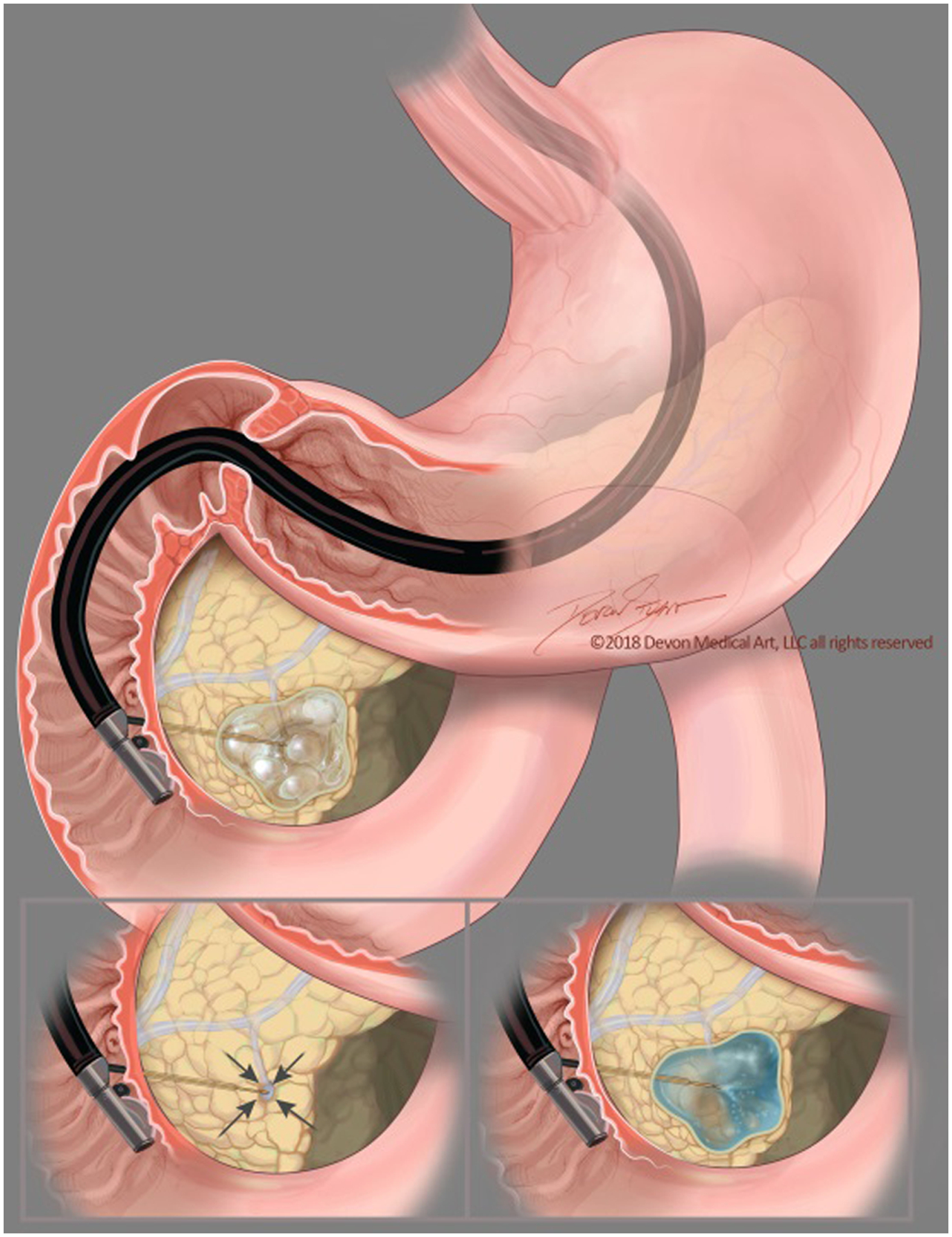
The EUS-guided cyst ablation process: The FNA needle is introduced into the center of the cystic lesion. Following near complete aspiration of mucinous fluid from all compartments, the cyst is then repeatedly filled and aspirated with the lavage solution, always leaving a small rim of fluid around the needle tip to prevent damaging the cyst wall. This is followed by near complete aspiration of the lavage agent with subsequent filling with the chemoablation agent(s) using the same volume as was originally aspirated. If an alcohol-free ablation technique is used, there is the option to skip the lavage step and simply aspirate the mucinous fluid to near total collapse, subsequently filling the cyst with an equal volume of chemotherapy ablation agent(s)

Preparation for pancreatic cyst ablation is similar to standard EUS-FNA. Discussion with the anesthesiologist is important to ensure the patient will be relatively still during the actual ablation. If excessive movement such as coughing, retching, or excessive respiratory movement is felt to be likely, general anesthesia with paralysis should be considered. The ablative agents, including any lavage agent used and the chemotherapy infusion, (i.e., paclitaxel or paclitaxel + gemcitabine) should be prepared prior to the procedure. The use, ordering, delivery, and disposal of chemotherapy ablation agents should be standardized at each individual institution. After a full endosonographic evaluation, the FNA needle is introduced into the center of the cyst, carefully aspirating nearly the entire cyst contents. It is our practice to use a 22-gauge FNA needle for a cyst measuring 2–3 cm in diameter and reserving 19 gauge needles for cysts > 3 cm in diameter or previously known to have highly viscous cyst fluid. If alcohol lavage is used, the cyst will be then alternately filled and aspirated with ethanol for 3–5 min using an amount equal to the mucinous fluid originally aspirated. A small rim of fluid around the needle tip is recommended at all times to avoid damaging the cyst wall with the needle tip and ensuring the injectate remains in the cyst (Fig. 1). If an alcohol-free approach is used, the lavage step of the procedure may be eliminated, and instead after initial cyst collapse, the chemoablation agent is then infused into the cyst using an amount equal to the volume of cyst fluid originally aspirated to refill the cyst to its original dimensions. As the paclitaxel is generally quite viscous, infusing the chemotherapy agent(s) typically requires high-pressure and so an infusion apparatus, such as a syringe strapped to a high-pressure gun or infusion device is often used [27••]. Antibiotics are recommended as per ASGE guidelines on the subject [33], and it has been our approach to observe these patients for an additional time post-operatively.

#### Post-Operative Care and Follow-Up

**Fig. 2:**
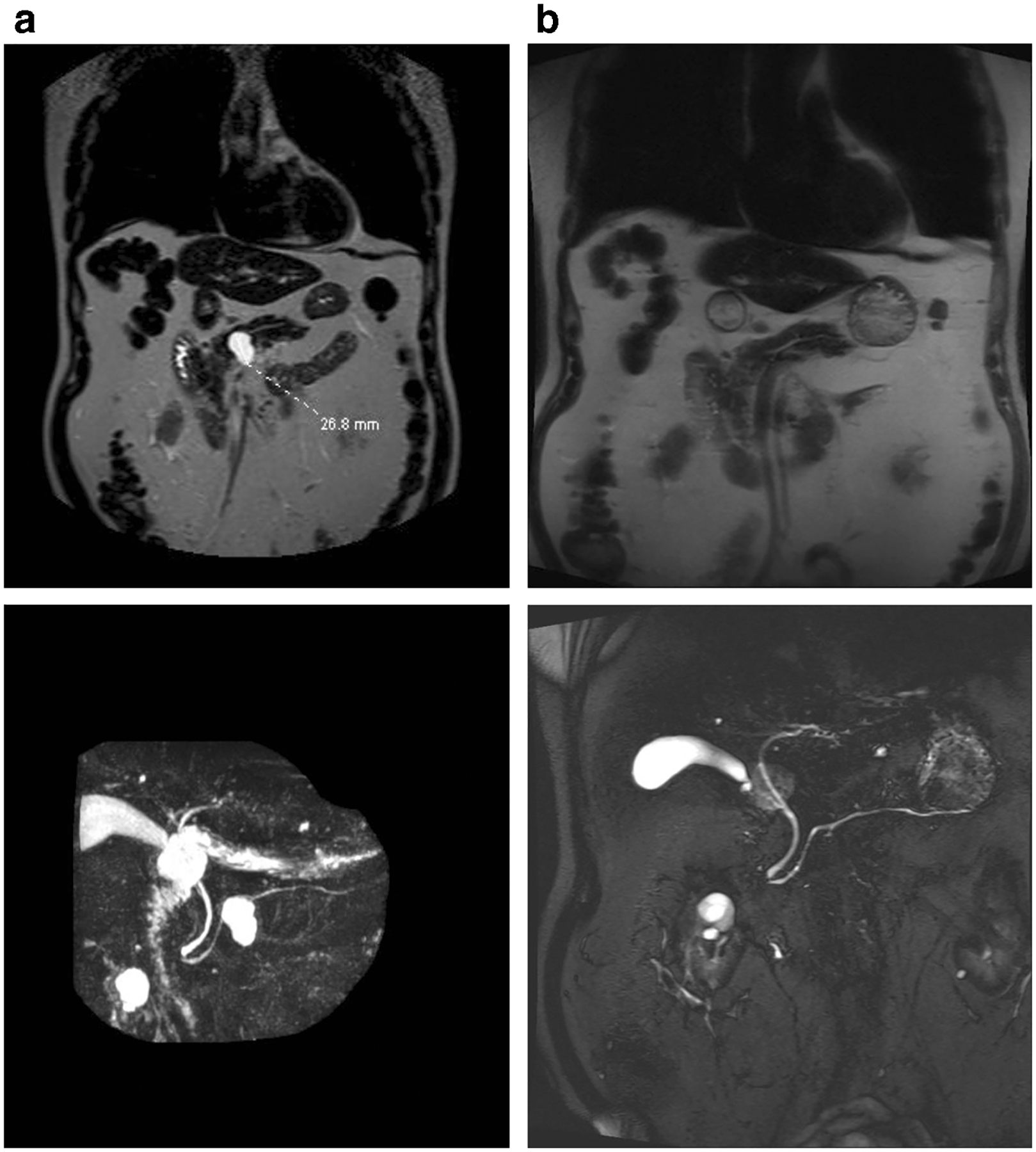
**a** MRI-MRCP imaging of an 83-year-old male with a 2.7-cm intraductal mucinous pancreatic neoplasm (white arrow) prior to EUS-guided chemoablation. **b** Follow-up MRI-MCRP imaging reveals no evidence of residual cyst at 6 month follow-up evaluation

DiMaio et al. demonstrated that the use of serial ablation procedures (similar to what is done for dysplastic Barrett’s esophagus) is more effective than a single ablation session for pancreatic cystic tumors [21]. The approach at our institutions utilizes 2–3 alcohol-free, gemcitabine-paclitaxel infusions at 3-month intervals. Residual cysts at the second and third endoscopies measuring > 15 mm are retreated if required. This is followed by a clinic evaluation and follow-up cross-sectional imaging at 6 and 12 months with either enhanced MRI or CT scan to measure response and assess for complications (Fig. 2). The definition for treatment response has been standardized over multiple studies. Baseline cyst size is measured as a volume (4/3 × π × r^3^ where r is cyst radius). Treatment response defined as complete (≥ 95% reduction of cyst volume), partial (94–75% reduction), or non-response (< 75% reduction) at follow-up [16•, 25, 27••, 29]. At our programs, patients then re-enter a surveillance program using the post-treatment measurements and characteristics of the pancreatic cyst(s) to govern surveillance type and frequency as per guidelines on this subject [6, 7•, 30].

## Limitations and Areas of Uncertainty

EUS-guided pancreatic cyst ablation is a promising and emerging approach for treating a known precursor lesion to a lethal malignancy which has a poor prognosis and treatment options limited to surveillance or a morbid surgery. As an evolving approach, it has multiple areas of uncertainty and limitations which critics have noted [35, 36]. Several of the most common critiques are discussed below.

### 1. Since ablation does actually not remove the cysts, what evidence is there that this procedure will provide a durable treatment response, and what if the lesion recurs?

This is similar to the previous concerns raised regarding RFA for dysplastic Barrett’s esophagus when critics believed that only esophagectomy (also a morbid surgery) could assure that all dysplasia was effectively treated. To address this valid question, DeWitt, et al. reported a prospective trial of 22 patients with mucinous cysts who underwent alcohol ablation followed by paclitaxel infusion and followed for a median of 27 months. While only 50% of patients achieved complete ablation, those patients with complete ablation showed a durable effect over a 27-month median follow-up [26]. More recently, Choi et al. reported the results of 164 patients who underwent EUS-guided ablation with ethanol followed by paclitaxel. 72% of patients achieved complete ablation and over a 6-year follow-up period, 98.3% of patients who achieved complete ablation remained in complete remission at follow-up [29]. The authors concluded this treatment approach is an effective and durable alternative to surgery. Overall, while no ablative strategy in gastroenterology can be expected to be completely effective, EUS-guided pancreatic cyst ablation appears durable when complete ablation is achieved.

### 2. Ablation does not completely eliminate a patient’s risk of developing pancreatic adenocarcinoma.

Large-scale studies demonstrating a reduction in the risk of developing adenocarcinoma after mucinous pancreatic cyst ablation are lacking, resulting in an area of needed research. For example, the near complete epithelial ablation of a progressively growing 3-cm mucinous cystic neoplasm likely significantly reduces but does not completely eliminate the chance of developing pancreatic malignancy. Two large retrospective, multicenter studies in Japan demonstrated that in patients under long-term surveillance for IPMN’s, some of which had been treated surgically, there was a 2–4% risk of developing a metachronous pancreatic malignancy in the remaining pancreas [37, 38]. Additionally, recent evidence shows an ongoing and progressive risk of malignant transformation in IPMN’s > 1 .5cm during long-term surveillance [32]. Consequently, whether a surgical, ablative, or surveillance strategy is employed, current guidelines suggest that long-term surveillance will be required until age and/or co-morbidities of the patient make further surveillance unwarranted. An exception would be that patients with surgically resected mucinous cystic neoplasms (MCN variant) may not require long-term radiographic surveillance [6, 7•].

Although it is important to critique any novel treatment approach, it is likely not reasonable to demand that EUS-guided ablation must achieve complete radiologic response or epithelial ablation in all patients to be considered a viable treatment option. Indeed, 100% efficacy is not achievable in other well-accepted ablative strategies in gastroenterology. For example, treatment of dysplastic Barrett’s esophagus with radiofrequency ablation has a known efficacy for complete eradication of metaplasia, high- and low-grade dysplasia of 77, 81, and 90% with disease recurrence rates of 32% and a 1% risk of developing invasive carcinoma despite completing treatment [39–41]. Recent studies report that colonoscopy is less effective in preventing proximal colon cancer death with impaired protection against right-sided colorectal cancer likely due to multiple factors, and with a known miss rate of 17% for polyps over 1 cm [42–44]. In fact, even surgical resection of pancreatic cysts does not completely eliminate the risk of malignancy, as a 2–4% risk of developing adenocarcinoma would remain in the residual pancreas and would continue to increase according to long-term follow-up studies. This suggests that that any treatment strategy will require long-term surveillance, as long as the age and co-morbidities of the patient indicate that this is appropriate [6, 32, 33, 37, 38].

### 3. Pancreatic cyst ablation is dangerous.

The overall risk of adverse event rates of EUS-guided ablation have historically ranged between 2 and 10%, with pancreatitis, peritonitis, venous thrombosis, and abdominal pain described. In previous trials, nearly all of these complications have been blamed on the inflammatory and toxic effects of alcohol. However, recent randomized prospective data indicates that alcohol is not required for effective pancreatic cyst ablation when a chemotherapy cocktail specifically designed for pancreatic neoplasia is used. More importantly, when alcohol is eliminated from the ablation process, adverse events rates similar to that seen in EUS-FNA are achieved. For this reason, evidence now indicates that EUS-guided cyst ablation should be, in our opinion, approached with an alcohol-free chemoablation technique, with or without lavage, using paclitaxel alone or with a gemcitabine-paclitaxel admixture. This combination has been demonstrated to have equal efficacy and significant improvement in safety over alcohol-based chemoablation. Additionally, multiple trials have now shown that there are undetectable blood levels of chemotherapy agent during the process of pancreatic cyst ablation which was originally a concern [27••, 45].

## Conclusions

EUS-guided pancreatic cyst ablation is a promising and emerging minimally invasive technique for the elimination of a known precursor lesion to a lethal malignancy which currently has poor therapeutic options limited to either a morbid surgery or indefinite surveillance. Careful patient selection is required, as is careful attention to the technical aspects of the procedure, post-procedure evaluation, and follow-up surveillance. In our experience, patients are almost universally in favor of eliminating an appropriate neoplastic-type pancreatic cyst and overwhelmingly favor a minimally invasive approach to do so. An emerging body of literature demonstrates the efficacy of appropriately performed pancreatic cyst ablation, a durable effect when a complete response is achieved, and a significantly improved safety profile with an alcohol-free protocol. Where cyst ablation fits into current treatment algorithms should be an ongoing discussion based on efficacy, safety, and the risk-benefit ratio unique to that patient. Additionally, EUS-guided ablation may offer a favorable cost profile when compared to EUS-FNA with molecular testing, Whipple surgery, or surveillance MRI-MRCP.

There are several areas of limitations and uncertainty with this technique. Most notably, there are no trials yet which demonstrate that this approach objectively reduces a patient’s progression to pancreatic adenocarcinoma. This emerging technique represents an exciting treatment option for appropriately selected patients; however, further studies are required to further develop the efficacy, safety, and treatment indications and to define which patients are best offered this emerging treatment option.

## Figures and Tables

**Table 1:** Summary of EUS-guided ablation trials: ethanol, ethanol + paclitaxel, alcohol vs. gemcitabine + paclitaxel. Italics = EtOH only. Bold = EtOH + paclitaxel. Bold italics = EtOH + paclitaxel–gemcitabine vs. saline + paclitaxel–gemcitabine

Author, year	Study type	Conditions (no. of patients)	Complete (CR) or partial resolution (PR)	Clinically significant adverse events^a^
*Gan et al. 2005* [18]	*Prospective (pilot)*	*5–80% EtOH (25)*	*35% CR* *7% PR*	*0%*
*Dewitt et al. 2009* [20•]	*Prospective (RCT)*	*80% EtOH (25)* *Saline (17)*	*33% CR* ^*2*^ *0% CR*	*24% (4% Pancreatitis, 20% Ab pain)* *12% (Ab pain)*
*DiMaio et al. 2011* [21]	*Retrospective*	*80% EtOH (13)*	*38% CR*	*8% (Ab pain)*
*Caillol et al. 2012* [19]	*Retrospective*	*99% EtOH (13)*	*85% CR*	–
*Gomez et al. 2016* [22]	*Prospective (pilot)*	*80% EtOH (23)*	*9% CR* *44% PR*	*8% (4% Pancreatitis, 4% Ab pain)*
**Oh et al. 2008** [23]	**Prospective**	**88–99% EtOH + paclitaxel (14)**	**79% CR** **14% PR**	**21% (7% Pancreatitis, 14% Ab pain)**
**Oh et al. 2009** [24]	**Prospective**	**99% EtOH + paclitaxel (10)**	**60% CR** **20% PR**	**10% (Ab pain)**
**Oh et al. 2011** [25]	**Prospective**	**99% EtOH + paclitaxel (47)**	**62% CR** **13% PR**	**4% (2% Pancreatitis, 2% Ab pain)**
**DeWitt et al. 2014** [26]	**Prospective**	**100% EtOH + paclitaxel (22)**	**50% CR** **25% PR**	**23% (10% Pancreatitis, 13% Ab pain)**
***Moyer et al. 2017*** [27••]	***Prospective (RCT)***	***80% EtOH paclitaxel + gemcitabine (18)*** ***Saline + paclitaxel + gemcitabine (21)***	***61% CR*** ***67% CR***	***28% (6% Pancreatitis, 22% Ab pain)*** ***0%***

a The overall % of adverse events described here represents the sum of AEs reported in corresponding studies (in parentheses), focusing on the two most common AEs reported—abdominal pain and pancreatitis. However, based on reported study results, it cannot be determined whether AE categories overlapped (e.g., whether a patient documented with pancreatitis also was a reported AE rate for abdominal pain). Other less commonly reported AEs include intracystic bleeding (26), splenic vein obliteration (21), development of an inflammatory gastric wall cyst (27), and peritonitis (27)
